# Potentially Zoonotic Bacteria in Exotic Freshwater Turtles from the Canary Islands (Spain)

**DOI:** 10.3390/biology14121753

**Published:** 2025-12-06

**Authors:** Román Pino-Vera, Néstor Abreu-Acosta, Oscar Afonso, Pilar Foronda

**Affiliations:** 1 Instituto Universitario de Enfermedades Tropicales y Salud Pública de Canarias, Universidad de La Laguna (ULL), Av. Astrofísico F. Sánchez, sn, 38203 La Laguna, Canary Islands, Spain; rpinover@ull.edu.es (R.P.-V.); nabreu@ull.edu.es (N.A.-A.); 2 Departamento de Obstetricia y Ginecología, Pediatría, Medicina Preventiva y Salud Pública, Toxicología, Medicina Legal y Forense y Parasitología, Facultad de Farmacia, Universidad de La Laguna (ULL), Av. Astrofísico F. Sánchez, sn, 38203 La Laguna, Canary Islands, Spain; 3 Programa de Doctorado de Ciencias Médicas y Farmacéuticas, Desarrollo y Calidad de Vida, Universidad de La Laguna (ULL), Av. Astrofísico F. Sánchez, sn. (Edificio Calabaza), 38200 La Laguna, Canary Islands, Spain; 4 Programa de Doctorado en Biodiversidad y Conservación, Universidad de La Laguna (ULL), Av. Astrofísico F. Sánchez, sn. (Edificio Calabaza), 38200 La Laguna, Canary Islands, Spain; oafopon@gesplan.es; 5 Área de Medio Ambiente Las Palmas, Cambio Climático y Proyectos Europeos, Gestión y Planeamiento Territorial y Medioambiental, S.A. GESPLAN, Av. Tres de Mayo, 71, 38005 Santa Cruz de Tenerife, Canary Islands, Spain

**Keywords:** Canary Islands, zoonoses, freshwater turtles, invasive species, bacteria

## Abstract

The pond slider (*Trachemys scripta*) is native to the southeastern United States but has been introduced all around the world, including to the Canary Islands (Spain), along with other less-common exotic freshwater turtles such as *Graptemys pseudogeographica*, *Mauremys* spp., and *Pseudemys peninsularis*. The aim of this study was to determine the presence of pathogenic bacteria in these animals and to evaluate the associated health risks for humans and local fauna. For this purpose, cloacal samples from 42 specimens collected on the islands of Tenerife and Gran Canaria (Canary Islands) were analyzed for potentially zoonotic bacteria using selective culture media and PCR. Non-tuberculous mycobacteria were the most isolated pathogen (57.9%), followed by *Yersinia enterocolitica* (42.1%) and *Escherichia coli* carrying *stx* and/or *eae* genes (33.3%). *Salmonella* spp. was detected in 31.0% of the chelonians, identifying *Salmonella* Typhi and *Salmonella* Typhimurium serotypes. *Staphylococcus* spp. showed a prevalence of 21%, mainly *Staphylococcus aureus* along with one antibiotic-resistant *Staphylococcus hominis* isolate. *Pseudomonas* spp. were found in 10.1% of samples, although only one isolate corresponded to *Pseudomonas aeruginosa*. *Campylobacter* spp. and *Vibrio* spp. were detected at low frequencies (<10%), and *Listeria monocytogenes* was not identified. Overall, the results indicate that aquatic turtle populations in the Canary Islands pose notable health risks, especially for animal handlers and people with compromised immune systems.

## Simple Summary

Pond sliders are native to the southeastern United States, but they can be found all around the world. In the Canary Islands (Spain), they coexist with other, less-common exotic freshwater turtle species, most likely as a result of being released from captivity. The aim of this study was to determine the presence of potentially pathogenic bacteria in these species, collected on the islands of Tenerife and Gran Canaria (Canary Islands), and to assess the associated health risks for humans and local fauna. The results indicate that the reptile populations examined carry bacteria that can be pathogenic to both humans and other animals and are mainly associated with gastrointestinal disease. These pathogens pose a particular risk to children and the elderly, while animal handlers and pet owners constitute the most exposed groups.

## 1. Introduction

The World Health Organization (WHO) defines zoonoses as “any disease or infection that is naturally transmissible from vertebrate animals to humans” [1]. Zoonotic viruses, bacteria, fungi, and parasites can be transmitted through direct contact or through food, water, vectors, or the environment [2], contributing to 61% of human infections, or even more in the case of emerging and reemerging diseases, with approximately 75% of them being related to animals [3,4]. Zoonotic diseases are not limited to rural areas and can also appear in urban settings, even if there are not noticeable animal populations in the surroundings, because of food and water contamination [5]. While zoonotic pandemics have been affecting humans since neolithic times, since humanity started to domesticate animals and plants, their relevance to human health has been particularly highlighted by recent highly virulent infections with pandemic potential, such as the 2005 H5/N1 avian influenza outbreak, the 2009 “swine flu” H1/N1 influenza pandemic, the 2013–2016 West African Ebola outbreak, and the 2019 coronavirus disease (COVID-19) pandemic, as well as local outbreaks of “neglected zoonoses” that can also have significant consequences [6,7].

Related to this, the introduction of foreign species into new habitats, mainly caused by the live animal trade stimulated by the growing tendency of owning exotic pets, increases the risk of zoonotic infections that can be life-threatening, particularly in children and immunocompromised people [8,9]. Some of these infections could be incorrectly prevented, diagnosed, or threatened because of the lack of knowledge of local health systems, which could have insufficient experience or training with these kinds of diseases [10,11]. Native fauna can also be infected with pathogens carried by introduced species, as well as being affected by predation, competition, and habitat alteration [12], especially species that belong to insular territories, due to the lack of competitors and predators that facilitate the settlement of invasive species and the spread of zoonotic diseases [13,14].

The Canary Islands (Spain) are located in north-west Africa, near the Morocco coastline (13°23′–18°80′ W and 27°37′–29°24′ N), and present the ideal life conditions for exotic species: warm temperatures, high availability of resources, and absence of predators. For these reasons, the archipelago harbors more than 340 invasive (or potentially invasive) animal and plant species, such as the barbary ground squirrel (*Atlantoxerus getulus*), the rose-ringed parakeet (*Psittacula krameri*), and the crimson fountaingrass (*Cenchrus setaceus*) [15,16]. Exotic reptiles are also present in the Canaries, the main example being the California kingsnake (*Lampropeltis californiae*), which has decreased the population of endemic lizards due to its dietary habits, and has been recognized as a carrier of zoonotic bacteria and parasites [17,18,19]. Moreover, exotic freshwater turtles have also been found on the islands, mostly pond sliders (*Trachemys scripta*) [20]. This chelonian, included in the Spanish catalog of invasive exotic species [21], is native to the southeastern United States and is the most widely distributed species in comparison with its endemic area, being found all around the world except for Antarctica [22]. In general, pet turtles can carry different bacteria without showing any clinical signs and shed them through their feces, posing an emerging public health concern [23]. However, despite all the investigations conducted on *Salmonella* in reptiles, which is the most contracted pathogen from this animal group, the presence and prevalence of other bacteria remain unclear [24]. Even though the zoonotic risk of other exotic reptiles found in the Canary Islands has been studied [19,25,26], there is no research regarding the microflora of wild freshwater turtles in this territory. For this reason, the aim of this study was to determine the presence of pathogenic bacteria in these animals and to evaluate their health risk to humans and local fauna.

## 2. Materials and Methods

### 2.1. Study Area and Specimen Collection

**Figure 1:**
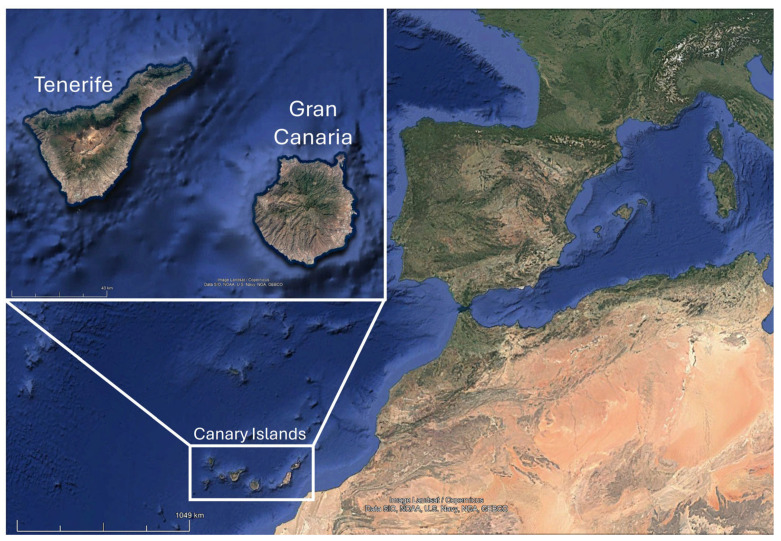
Location of Tenerife and Gran Canaria, where the specimens were collected. Image obtained from Google Earth Pro (v. 7.3.6.10441) and modified with Microsoft PowerPoint software (v. 2505).

A total of 42 adult freshwater turtles (2 *Graptemys pseudogeographica*, 7 *Mauremys* spp., 3 *Pseudemys peninsularis*, 30 *T. scripta*) from the islands of Tenerife and Gran Canaria (Figure 1) were euthanized, frozen, and donated by the staff of the “Red de Alerta Temprana de Canarias para la Detección e Intervención de Especies Exóticas Invasoras” (REDEXOS) during 2021–2023, affiliated with a company Gestión y Planeamiento Territorial y Medioambiental, S.A. (GESPLAN) (Santa Cruz de Tenerife, Spain). This action was carried out following authorization from the “Dirección General de Lucha Contra el Cambio Climático y Medio Ambiente, Gobierno de Canarias” (Las Palmas de Gran Canaria, Spain). Table 1 shows the species studied and the location of their capture. 

### 2.2. Sampling and Bacterial Isolation

The processing of the samples was performed after thawing the specimens at 4 °C and under aseptic conditions, using a class II biological safety cabinet (Telstar, Barcelona, Spain). Two cloacal samples were taken from each sampled animal using sterile swabs (Deltalab, Barcelona, Spain), and were incubated with 5 mL of Buffered Peptone Water (BPW) (Labkem, Barcelona, Spain). One sample was incubated at 37 °C for 24 h, and the other one at 42 °C for 18 h under microaerophilic conditions (using a 5 mL Eppendorf tube to reduce the oxygen percentage) for *Campylobacter* spp. isolation. An additional sample was obtained under the same conditions and stored for 8 h at 37 °C in 5 mL of Alkaline Peptone Water (APW) (1% NaCl, pH = 8.4) for *Vibrio* spp. Subsequently, 100 µL of liquid culture were inoculated onto different selective culture media: Baird–Parker agar (Labkem) for *Staphylococcus* spp., Cetrimide agar (VWR International, Leuven, Belgium) for *Pseudomonas* spp., Cefsulodin Irgasan Novobiocin agar (CIN) (Merck, Darmstadt, Germany) for *Yersinia enterocolitica*, Oxford agar for *Listeria monocytogenes* (Labkem), Thiosulfate citrate bile salts sucrose agar (TCBS) (VWR International) for *Vibrio* spp., sorbitol supplemented MacConkey agar (Scharlab, Barcelona, Spain), and Tryptone Bile X-glucuronide chromogenic agar (TBX) (Labkem) for *Escherichia coli*. Every plate was incubated for 24 h at 37 °C, except for the CIN, which was stored at 30 °C. In the case of *Salmonella* spp., 500 µL of BPW culture was transferred to 4.5 mL of Rappaport–Vassiliadis broth (VWR International) and incubated for 20 h at 42 °C. Then, 100 µL of the liquid culture was later incubated in *Salmonella*–*Shigella* agar (Merck) for 24 h at 37 °C.

### 2.3. Molecular Biology Techniques

#### 2.3.1. DNA Extraction

The colonies obtained were suspended in 1 mL of PBS under sterile conditions, followed by centrifugation at 12,000× *g*; then, the supernatant was discarded, and the process was repeated again. The resulting pellet was subjected to DNA extraction following López et al.’s [27] protocol. The same methodology was applied for the DNA isolation of *Mycobacterium* spp. and *Campylobacter* spp. using 1 mL of each BPW culture.

#### 2.3.2. PCR Identification

Different polymerase chain reaction (PCR) techniques were performed for the identification of relevant zoonotic bacteria, along with resistance and virulence genes:

Six pairs of primers were employed for *Campylobacter* spp. (23s rRNA fragment) confirmation and *Campylobacter coli* (*glyA* gene), *Campylobacter fetus* (*sapB2* gene), *Campylobacter jejuni* (*hipO* gene), *Campylobacter lari* (*glyA* gene), and *Campylobacter upsaliensis* (*glyA* gene) identification, according to Wang et al. [28].

Following the protocol described by Blanco et al. [29], some *E. coli* pathotypes were identified through the amplification of *stx*_1_, *stx*_2_, and *eae* virulence genes, responsible for Shiga-like toxins and intimin protein synthesis, respectively.

*Listeria monocytogenes* that grew in Oxford agar was identified by a simple PCR of a region of the *iap* gene, which codifies the p60 invasion-associated protein, as described by Jaton et al. [30].

Mycobacteria identification was carried out using a multiplex PCR described by Kim et al. [31]. This protocol also allows the differentiation between the *Mycobacterium tuberculosis* complex and the atypical mycobacteria group by amplifying the *rpoB* gene, and the regions of difference (RD) RD1 and RD8.

The colonies from cetrimide agar were irradiated with UV light, and the fluorescent ones were tested for *Pseudomonas aeruginosa* through simultaneous amplification of lipoprotein-coding genes: *oprI* and *oprL*, as described by De Vos et al. [32].

The identification of *Salmonella enterica* serotypes important to human health was carried out following De Freitas et al.’s [33] protocol. Two different PCRs were performed: one to identify *Salmonella* Enteritidis (*sdfI* gene) and *Salmonella* Typhi (*ViaB* gene), and a second one for *Salmonella* Typhimurium detection (*Spy* gene). In both cases, *Salmonella* spp. (*OMPC* gene) was tested.

A single m-PCR described by Campos-Peña et al. [34] was used for the identification of six *Staphylococcus* species: *Staphylococcus aureus* (*nucA* gene), *Staphylococcus epidermidis* (*sep* gene), *Staphylococcus haemolyticus* (*mvaA* gene), *Staphylococcus hominis* (*hom* gene), *Staphylococcus lugdunensis* (*fbl* gene), and *Staphylococcus saprophyticus* (*sap* gene), as well as for the detection of methicillin (*mecA* gene) and mupirocin (*ileS2* gene) resistance genes. 

According to Liu et al. [35], a PCR assay was performed to detect all bacteria belonging to the *Vibrio* genus by amplifying a fragment of 16s rDNA. A more specific PCR described by Neogi et al. [36] was performed with the positive samples, based on *toxR* gene amplification to identify *Vibrio cholerae* and *Vibrio parahaemolyticus*, and the *vvhA* gene for *Vibrio vulnificus*.

The colonies grown in CIN agar were tested for the *ail* (attachment and invasion locus) gene to identify pathogenic and non-pathogenic *Y. enterocolitica* strains, according to Wannet et al. [37].

All PCR assays were evaluated with 1.5% agarose gel electrophoresis (Fisher Bioreagents, Madrid, Spain) at 90 V for 1 h. SiZer-100 DNA Marker (iNtRON Biotechnology, Seongnam-Si, Republic of Korea) was used as molecular size marker and Real-Safe (Durviz SL, Valencia, Spain) as DNA stain. The gels were revealed with a ChemiDocTM XRS+ (Bio-Rad, Hercules, CA, USA) system.

#### 2.3.3. Controls

Positive controls were employed in all PCR assays, using bacterial strains from the American Type Culture Collection (ATCC). These strains were stored at −70 °C and incubated for growth for 18 to 24 h in Tryptic Soy Broth (TSB) (Labkem) at 37 °C under aerobic conditions, or microaerophilic conditions in the case of *Campylobacter* spp. Subsequently, they were submitted to DNA extraction using the same method used for the samples. For the negative controls, nuclease-free molecular biology grade water (VWR International) was used instead of DNA.

### 2.4. Statistical Analysis

The chi-square test and Fisher’s exact test were applied, establishing a *p*-value of 0.05, to compare the prevalence between the turtle species and the islands where the studied animals were found. This was performed using the statistical Windows software “Statistical Package for the Social Sciences” (SPSS) 29.0.1.0 (IBM Corporation, Armonk, NY, USA). The 95% Clopper Pearson confidence intervals (95% CI) were calculated using the approximate or exact method, as appropriate.

## 3. Results

### 3.1. General

*Mycobacterium* spp. was the most isolated pathogen in the forty-two turtles studied, being identified in eleven out of nineteen animals (57.9%; 33.5–79.7), followed by *Y. enterocolitica* in eight out of nineteen (42.1%; 20.3–66.5), and virulent *E. coli* in fourteen out of forty-two (33.3%; 19.6–49.5). In contrast, none of the thirty-six turtles tested for *L. monocytogenes* showed positive results. Table 2 summarizes all positive results isolates.

### 3.2. Campylobacter spp.

None of the most clinically relevant *Campylobacter* species (*C. coli*, *C. fetus*, *C. jejuni*, *C. lari*, and *C. upsaliensis*) were detected in this study; however, four isolates were identified at the genus level. Three of them were from Gran Canaria (one *P. peninsularis* and two *T. scripta*) and one from *T. scripta* from Tenerife, with no statistical differences observed between the islands.

### 3.3. Escherichia coli (stx_1_, stx_2_ and Eae Genes)

Virulent *E. coli* genes were detected in fourteen out of forty-two turtles (33.3%; 19.6–49.5). The most prevalent gene was *stx*_2_, found in seven animals (16.7%; 7.0–31.4), followed by *eae* and *sxt*_1_ being found in six (14.3%; 5.4–28.5) and four (9.5%; 2.7–22.6) turtles, respectively. Three *T.scripta* showed the coexistence of two different genes: *eae* + *sxt*_1_ (Tenerife), *eae* + *stx*_2_ (Gran Canaria), and *sxt*_1_ + *stx*_2_ (Tenerife). Detailed data are shown in Table 3. There were no statistical differences between the prevalences of turtle species or island.

### 3.4. Listeria monocytogenes

Thirty-six turtle samples were tested for *L. monocytogenes*, and all tested negative.

### 3.5. Mycobacterium spp.

The exclusive amplification of the *rpo*B gene in eleven out of nineteen (57.9%; 33.5–79.7) turtles evidences the presence of atypical (non-tuberculous) mycobacteria in these specimens. No significative differences were found between the prevalences by species or island. Detailed results are shown in Table 4.

### 3.6. Pseudomonas spp.

*Pseudomonas* spp. was detected in two out of nineteen (10.1%; 1.3–33.1) animals tested (*T. scripta* from Tenerife) with no statistical differences between species or islands. The amplification of both *oprI* and *oprL* genes indicated the presence of *P. aeruginosa* in one (5.3%; 0.1–26.0) of them.

### 3.7. Salmonella spp.

*Salmonella* spp. was detected in thirteen out of forty-two (31.0%; 17.6–47.1) turtles. More specifically, *S*. Typhi and *S*. Typhimurium serotypes were found coinfecting one (2.4%, 0.06–12.6) *T. scripta* from Tenerife, but *S*. Enteritidis were not identified. Results are shown in Table 5. No statistical differences were found between the prevalences by turtle species or island.

### 3.8. Staphylococcus spp.

Nine positive isolates were obtained for *Staphylococcus* spp. from the forty-two chelonians studied (21.4%; 10.3–36.8), with *S. aureus* being identified in eight cases (19.0%; 8.6–34.1). The remaining isolate (2.4%; 0.06–12.6), from one *T. scripta* from Tenerife, was characterized as mupirocin-resistant *S*. *hominis*. All results are shown in Table 6. No statistical differences were observed between the prevalences by species or island.

### 3.9. Vibrio spp.

Thirty-six animals were tested for *Vibrio* sp., yielding positive results in one *P. peninsularis* and one *T. scripta* from Gran Canaria (5.6%; 0.7–18.7); however, a posterior PCR resulted negative for *V. cholerae*, *V. parahaemolyticus*, and *V. vulnificus*.

### 3.10. Yersinia enterocolitica

Eight out of nineteen turtles tested for *Y. enterocolitica* were positive for this bacterial species, with no statistical differences between islands. The presence of *ail* gene was not observed in any sample. Table 7 shows detailed results.

## 4. Discussion

### 4.1. Campylobacter spp.

Some *Campylobacter* species are well-known zoonotic agents with importance for human and veterinary health. The gastrointestinal disease they cause (campylobacteriosis) is one the most common bacterial illnesses and its incidence has been increasing over the last decade, causing symptoms like fever, abdominal pain, vomiting, diarrhea, and, in fewer cases, extraintestinal infections and/or autoimmune disorders [38,39]. *Campylobacter jejuni* and *C. coli* are the most frequent species that cause human infection, which are part of the microbiome that infect warm-blooded animals such as pigs and poultry, and are normally asymptomatic. For this reason, the main entry way for *Campylobacter* spp. into hosts is through the host’s consumption of contaminated animal products [40]. Other species, such as *C. upsaliensis* or *C. lari*, have been found to cause human disease; however, their actual clinical importance remains unknown because the specific *Campylobacter* species involved are not usually identified [41]. Four species have been isolated from reptiles to date: *C. fetus*, *Campylobacter geochelonis*, *Campylobacter hyointestinalis*, and *Campylobacter iguaniorum*, of which only *C. fetus* has been associated with human disease [42].

In our study, four (9.5%) *Campylobacter* spp. isolates were obtained, but none could be identified at the species level using our PCR protocol, which was designed for bacteria commonly involved in human campylobacteriosis. They may correspond to the previously mentioned reptile-associated species: *C. geochelonis*, *C. hyointestinalis*, and *C. iguaniorum*. Recent investigations have identified other species as responsible for disease [43,44], suggesting that the risk for humans and warm-blooded animals in contact with this turtle population could be higher. In general, the presence of *Campylobacter* in turtles is low, with reported prevalences ranging from 10.4% [45] to 1.1% or there even being a complete absence [46,47], which aligns with the results obtained in the Canary Islands. An exception is the study by Gilbert et al. [48] who reported 60.4% positive samples using PCR as the detection method. These authors noted that such differences could be due to the varying isolation and identification methods used in each study (specific media or PCR), as well as the intermittent shedding of microorganisms.

### 4.2. Escherichia coli (stx_1_, stx_2_ and Eae Genes)

*Escherichia coli* is widely distributed among vertebrates, especially warm-blooded animals and reptiles, showing different prevalences between species and being remarkably high in humans compared to reptiles [49]. Although *E. coli* is an important component of the intestinal microflora, its genetic plasticity has allowed the acquisition of multiple virulence factors, leading to different pathotypes that can cause both intestinal and extraintestinal infections, the latter mainly occurring in the urinary tract, but occasionally resulting in meningitis or endocarditis [50,51,52]. The *stx* and *eae* genes are responsible for a large part of the virulence of these pathogenic *E. coli* strains [53]; the first ones encode for verotoxins or Shiga-like toxins, divided into *stx*_1_ and *stx*_2_ with various subtypes each, and are related to bloody diarrhea and the life-threating hemolytic–uremic syndrome, especially *stx*_2_ [54,55]. Furthermore, the *eae* gene encodes for intimin, a protein that facilitates the attachment of *E. coli* to the intestinal epithelium, which is necessary for colonization [56].

Not many studies have tested reptiles for *E. coli* due to their association with warm-blooded animals, and even fewer have addressed its virulence [57]. The scarce data available show a low presence of Shiga-like *E. coli* (STEC) and/or intimin; for instance, Dec et al. [58] identified 32 out of 67 (47.8%) positive samples for *E. coli* among turtles, lizards, and snakes from Poland (with significantly similar prevalences between groups), half of which showed virulence factors, but none contained *stx*_1_, *stx*_2_, or *eae* genes. Martinez et al. [59] analyzed 20 ocellated lizards (*Timon lepidus*) from Spain without describing any positive STEC sample, and Bautista-Trujillo et al. [60] found low prevalences of virulent *E. coli* in 240 green iguanas (*Iguana iguana*) sampled in Mexico: 10% *stx*_1_, 0.4% *stx*_2_, and 0.8% *eae*. The results of our study are considerable higher than these and could suggest that, although reptiles are not major carriers of zoonotic *E. coli*, freshwater turtles from the Canary Islands could suppose a risk. However, further research is needed to confirm this hypothesis, along with the identification of other virulent factors and the characterization of the strains involved and their adaptability to the human host.

### 4.3. Listeria monocytogenes

*Listeria monocytogenes* is an opportunistic pathogen that mainly affects immunocompromised individuals, pregnant women and newborns, and can be found in soil, water, various food products, humans, and animals. This microorganism colonizes the intestinal tract through the ingestion of contaminated food and then disseminates to other organs, causing gastroenteritis, meningitis, encephalitis, mother-to-fetus infections, and septicemia, with a death rate of 25–30%. Even though listeriosis is rare compared to other foodborne infections, its high mortality makes this bacterium an important public health concern [61,62]. It is important to note that a cutaneous form of listeriosis can be contracted by veterinarians and farm workers from the animals they handle, and potentially spread the disease to their pets [63].

The adaptability of *L. monocytogenes* to different ranges of temperature, salinity, and pH allows its development in cold-blooded animals like reptiles [64]; even so, not many studies have been conducted on its prevalence in these animals, showing relatively low infection percentages. In wildlife animals from New York, Chen et al. [65] obtained a prevalence of 5.6% (18/324) overall, with the prevalence in reptiles (12%, 2/17) being slightly higher to mammals (8%, 5/64) and birds (4.5%, 11/242), while Nowakiewicz et al. [66] tested 130 European pond turtles (*Emys orbicularis*) from Poland, finding just two cases of *L. monocytogenes* in adults (1.5%, 2/130). The authors of these works comment that the observed differences could be due to the isolation methods applied. Of the 36 turtles tested in this study, none of them showed positive results for *L. monocytogenes*, suggesting that listeriosis infection may not be a major preoccupation to consider in freshwater turtles from the Canary Islands; nevertheless, further investigation is required to confirm this statement, taking into account the small sample size.

### 4.4. Mycobacterium spp.

Mycobacteriosis are a group of diseases with different symptomatology caused by various *Mycobacterium* species. These species are classified into the *M. tuberculosis* complex (primarily affecting the lungs) and non-tuberculous mycobacteria (NTM); these are also referred to as atypical or environmental mycobacteria because they can be isolated from water, soil, dust, and plants and frequently affect lymphatic, skin, and soft tissues [67,68,69]. Bacteria belonging to the atypical group (e.g., *Mycobacterium chelonae*, *Mycobacterium fortuitum*, *Mycobacterium kansasii*) can infect reptiles through cutaneous lesions or the ingestion of contaminated food and/or water; even so, reptiles appear to be naturally resistant, and in most cases are asymptomatic [24]. Many NTMs have been characterized as antibiotic-resistant, and despite not frequently affecting humans, case reports have been published; for this reason, children and individuals with compromised immune systems should take special precautions and avoid close contact with reptiles to minimize the risk of exposure [70].

While mycobacteriosis is more frequently reported in chelonians than in other reptile groups, mainly due their association with aquatic environments, most studies consist of case reports of sea turtles or, less frequently, freshwater turtles that show granulomatous lesions on viscera, bone or joint tissues, usually found postmortem [70]. In this study, non-tuberculous mycobacteria were detected in 11 out of 19 (57.9%) specimens. In contrast, the only study found searching for mycobacteria in wild freshwater turtles, conducted in Poland [71], reported a prevalence of 24.8% (31/125). This difference could be attributed to the warmer temperatures in the Canary Islands compared to Central Europe, which facilitates bacterial development, as well as the smaller sample size and the methodology employed [72,73]. Although our PCR protocol could not differentiate between species within this group, the most probable species present in the tested specimens is *M. chelonae*, as the most frequently identified mycobacterium in these animals, along with *Mycobacterium marinum* and *Mycobacterium haemophilum* [74,75]. Further studies should aim to identify the *Mycobacterium* species by amplifying and sequencing of other DNA fragments.

### 4.5. Pseudomonas spp.

Bacteria belonging to the *Pseudomonas* genus are known for their capacity to colonize a wide variety of environments, both aquatic and terrestrial, due to their metabolic and physiological adaptability [76]. Among all species, *P. aeruginosa* is the most extensively studied because of its pathogenic characteristics in plants and animals (including humans). It is often described as an opportunistic pathogen and is one of the most common causes of nosocomial infection, especially in individuals with compromised immune system, burns or wounds, or those using implants or indwelling medical devices [77,78]. In humans, *P. aeruginosa* can affect multiple organs including the skin, brain, eyes, ears, urinary tract, and lungs; however, urinary tract and pulmonary infections are the most common due to their ability to form biofilms on catheters and intubation equipment. Additionally, this bacterium possesses other virulence factors such as toxins, proteases, hemolysins, and antibiotic resistance mechanisms [79,80].

Regarding animals, *P. aeruginosa* can cause different symptomatology such as otitis in dogs, respiratory infections in cats, mastitis in cows or endometriosis in horses [81,82]. In reptiles, it is part of their oral and intestinal microflora but acts as an opportunistic pathogen too, although few reports have been published and these are focused mainly on lizards and snakes in which it causes ulcerative stomatitis, necrotizing enteritis, cloacitis, dermatitis, abscesses, and septicemia, among other symptoms [83]. The prevalence of *P. aeruginosa* found in this study (5.3%; 1/19) matches other works conducted in continental Spain such as Mengistu et al. [84], who found 2 out of 91 (2.2%) positive isolates from wild *T. scripta* samples, or Muñoz-Ibarra et al. [85], reporting 62 out of 345 (18.0%) positive reptiles (Testudines and Squamata) samples from Spain and Portugal; in this latter study, the authors analyzed other anatomical locations besides feces, like the skin or nose, which, along with the origin of the samples (from diseased animals belonging to clinics), might explain the higher percentage. In Italy, a study showed 9 positive *P. aeruginosa* isolates out of 218 (4.1%) healthy pet reptiles [86], suggesting that *P. aeruginosa* does not constitute a great threat to consider in freshwater turtles from the Canary Islands; however, more studies need to be conducted to affirm this hypothesis, considering the small sample size analyzed.

### 4.6. Salmonella spp.

*Salmonella* spp. is one of the most frequent causes of foodborne disease in humans, mainly through the consumption of poultry and eggs which are the primary sources of salmonellosis outbreaks [87]. Infections caused by this bacterium can be classified according to their pathogenicity: human-restricted serotypes (*S*. Typhi, *Salmonella* Paratyphi, and *Salmonella* Sendai) cause an invasive, life-threatening systemic disease known as typhoid or enteric fever, whereas nontyphoidal serotypes such as *S*. Enteritidis or *S*. Typhimurium normally cause self-limited gastroenteritis associated with intestinal inflammation and diarrhea lasting 5–7 days in immunocompetent individuals [88,89]. In animals, the most common clinical manifestation is a gastrointestinal disease, although acute septicemia, abortion, arthritis or respiratory disease can also be observed. However, infection often remain asymptotic, making control in farms and herds challenging [90].

Reptiles carry *Salmonella* spp. as part of their normal microbiota, with prevalences reaching up to 90% according to some studies, along with a wide variety of serotypes, some of them zoonotic [91]. This makes salmonellosis the most frequent zoonotic disease transmitted by pet reptiles [92]. In studies conducted on freshwater turtles from continental Spain, Hidalgo-Vila et al. [93,94] reported prevalences of 13,2% (10/76) and 6.6% (5/78) in free-living endemic turtles, and 6.38% (6/94) and 5.1% (2/39) in free-living and pet exotic turtles, respectively. They detected only one isolate of the zoonotic *S*. Typhimurium serotype in a single *T. scripta* turtle, with no presence of *S*. Typhi or *S*. Enteritidis. Marin et al. [95] reported that none of the 37 freshwater turtles (*E. orbicularis* and *Mauremys leprosa*) nor the 34 sea turtles (*Caretta caretta*) analyzed were positive for *Salmonella* spp. In contrast, tortoises tested in that study showed a prevalence of 36% (29/81), similar to the findings of Hidalgo-Vila et al. [93], where all samples (100%; 16/16) from *Testudo graeca* tortoises tested positive for this bacterial genus. The authors attributed these differences to the longer persistence of *Salmonella* spp. in terrestrial environments compared to aquatic ones, as well as to the geophagic and coprophagic habits of tortoises. 

The results of this study (31.0%; 13/42) are considerably higher compared to continental Spain, which could mean that the turtles analyzed were pets liberated by their owners or escaped from households where they cohabited with more turtles in the same terrarium, as can occur in zoos and especially in pet shops, which has been noted in different studies [96,97,98,99,100]. The finding of *S*. Typhi in one *T. scripta* specimen from Tenerife, a serotype considered human-restricted [101,102], indicates a contamination of the animal environment with human feces; also, the isolation of the zoonotic *S*. Typhimurium in the same specimen indicates the risk of infection to the surrounding human populations and, especially, animal handlers. More studies need to be carried out to identify other *Salmonella* serotypes in freshwater turtles from the Canary Islands.

### 4.7. Staphylococcus spp.

Many *Staphylococcus* species are part of the normal microflora of humans and animals (mostly skin and mucosa), traditionally divided according to their capacity to produce coagulase enzymes [103]. Staphylococci can be transmitted through direct contact with infected humans, animals or unclean sanitary equipment, as well as through ingestion of contaminated food or water [104], with *S. aureus* being the most studied species due to its virulence and antimicrobial resistance. In healthy humans, it predominantly colonizes the nose, throat, axillae, and groin, causing minor skin infections that do not usually require antibiotic treatment but, in hospitalized or immunocompromised patients, especially those with skin lesions, the severity of the infection is higher, producing abscesses, lung infections, bacteremia, endocarditis, or osteomyelitis, requiring antibiotic therapy; this makes *S. aureus* one of the most common causes of hospital-associated infection along with *P. aeruginosa* [105,106].

In warm-blooded animals, the infection is similar to that of humans; however, staphylococci in reptiles often produce cutaneous diseases as well as gastrointestinal ones, such as stomatitis, dental and liver disease, or cloacitis [107]. Most of the related bibliography uses skin reptile samples for *Staphylococcus* spp. identification, which cannot be directly compared to our study; even so, interesting results have been obtained. For example, Strompfová et al. [108] used the MALDI-TOF technique on 40 skin samples from 17 different reptile species and isolated 51 coagulase-negative staphylococci, mainly *Staphylococcus xylosus* (22/40; 55%) and *Staphylococcus sciuri* (16/40; 40%), both commensal of skin and mucosa with the second one identified as an occasional opportunistic pathogen; however, no *S. aureus* was identified. Using the same method, Brockmann et al. [109] analyzed skin samples from 235 different reptiles and found 25 (10.6%) positive *Staphylococcus* spp. samples. Of the few studies found that analyze fecal samples, one, conducted by Espinosa-Gongora et al. [110] in the Copenhagen Zoo, did not isolate any *S. aureus* in the 21 reptiles analyzed (chelonians, lizards and snakes), which was the same result as Almeida et al.’s study [111], where all 66 *Chelonoidis carbonaria* tortoises were negative for this bacterium; however, 48 (72.7%) showed positive results for coagulase negative staphylococci, mostly *S. sciuri* and *S. xylosus*. 

The notable difference in the prevalences of *S. aureus* in relation to our study (19.0%) indicates that the freshwater turtles from Tenerife and Gran Canaria could present a health risk to handlers, and especially, to people with a deficient immune system; however, more studies are needed to clarify the origin of the bacterium (e.g., contaminated water environment, contact with infected animals, etc.). One turtle was found carrying mupirocin-resistant *S. hominis*. This bacterium is typically found on human skin and rarely causes dermatological diseases, with some studies suggesting that it protects against the development of opportunistic pathogens [112,113]; therefore, its presence can indicate human contamination of the environment where the turtles inhabit. It is also important to mention that the PCR protocol used could lead to false negatives for less-common *Staphylococcus* species due to limited identification.

### 4.8. Vibrio spp.

The *Vibrio* genus comprises almost 200 described species that inhabit a wide range of aquatic environments [114,115]. Several species are pathogenic, with *V. cholerae*, *V. parahaemolyticus*, and *V. vulnificus* being the most important for human health; the first two cause gastroenteric disease with severe diarrhea (cholera), while the third causes wound infections and septicemia [116,117]. Vibriosis primarily occurs through the ingestion of raw or undercooked seafood contaminated with the bacteria or through wound contact with contaminated water, especially in warm seas [118,119]. Different *Vibrio* species have been detected in turtles; however, limited information is available regarding their symptomatology in these animals. They appear to be associated with skin and gastrointestinal lesions and may also cause bloodstream infection. Among the most common species identified in these reptiles, *Vibrio alginolyticus* can cause serious disease in humans, whereas *Vibrio harveyi* is an emerging opportunistic pathogen that affects many aquatic animals worldwide [120,121].

Studies focused on *Vibrio* spp. in turtles are mainly centered on sea turtles, particularly in Asia, where sea turtles are considered part of the human diet [122]. These studies show variable infection rates in cloacal samples, with *V. alginolyticus* and *V. parahaemolyticus* being frequently isolated, highlighting sea turtle consumption risk [121,123,124,125]. In this study, *Vibrio* prevalence was low, with only 2 positive turtles out of 36 (5.6%) being different from *V. cholerae*, *V. parahaemolyticus*, and *V. vulnificus*. This is probably due to *V. alginolyticus*’s reported abundance in similar animals; for this reason, more studies need to be conducted to precisely identify the species involved and determine the human infection risk.

### 4.9. Yersinia enterocolitica

Yersiniosis is a foodborne disease mainly caused by *Y. enterocolitica* that manifests as gastrointestinal inflammation, fever, vomiting, and diarrhea; it is especially intense in children under 5 years of age [126]. This bacterium can be found in a wide variety of animals and environments, with pigs (and pork products) being the main reservoir of human pathogenic strains; however, virulent serogroups have also been isolated in dogs, sheep, wild rodents, and water [127,128]. *Yersinia enterocolitica* is resistant to cold temperatures, allowing it to develop in refrigerated food, which is its main infection pathway [129]; despite this, not many studies have investigated its presence in cool-blooded animals.

The prevalence of *Y. enterocolitica* found in reptiles is considerably low: Silveira et al. [130] obtained one positive sample in 1 *Pantherophis guttatus* snake out of 23 (4.3%) wild reptiles from a rehabilitation center in Brazil; Nowakiewicz et al. [66] did not obtain any positive isolate from the 96 juvenile *E. orbicularis* turtles in a breeding center and only obtain positive isolate from 2 out of the 34 (5.9%) wild adult ones, both from Poland; and Kumar & Sharma [131] obtained negative results when analyzing 101 urban *Hemidactylus flaviviridis* geckos from India. These low results contrast with those obtained in this work (42.1%; 8/19); however, another study performed with exotic reptiles in the Canary Islands [26] obtained similar results, where 11 out of 29 (52.4%) veiled chameleons (*Chamaeleo calyptratus*) from Gran Canaria were positive for *Y. enterocolitica*, with only one isolate expressing the *ail* gene, which is typically found in pathogenic strains. In the case of the aquatic turtles, this gene was not detected; this absence may indicate an environmental origin and decrease the risk of human and animal infection.

### 4.10. Summary

The most frequently isolated bacteria were, in order of prevalence, *Mycobacterium* spp., *Y. enterocolitica*, and virulent *E. coli*. The detection of species and serotypes such as *S*. Typhi or *S. hominis*, which are considered specific or are mostly found in humans, may indicate close contact between the turtles and people, possibly due to contamination of the chelonian environment with wastewater from nearby households that, in some cases, are not connected to a sewerage system [132]. This finding could also suggest that some turtles captured in the wild had been released by their owners or had escaped from households where they were kept under poor livings conditions (i.e., overcrowded terrariums or unclean water supply). This would help explain the higher prevalence of some pathogens found, such as *Y. enterocolitica* or non-tuberculous mycobacteria, compared to previously published data. In addition to the risk to humans, the bacteria identified could affect other animals, even those far from the turtles’ habitat, through the contamination of watercourses.

Free-living turtles have been less studied than pet or zoological turtles, making it difficult to compare the results of this study with those of others. As far as is known, this is the first study conducted in the Canary Islands investigating the presence of zoonotic bacteria in wild freshwater turtles and providing relevant data on the public health risks posed by these species. However, the small sample size of some of the species examined, which limits statistical analysis, and the PCR methods employed, which did not allow to identify all the isolates obtained, constitute its main limitations. For these reasons, further studies should include larger numbers of specimens from different species, target additional pathogens or serotypes, and incorporate antibiotic resistance and virulence factor assays. It would also be useful to investigate the role of turtles in bacteria dissemination and to distinguish between reservoirs and mechanical carriers. The analysis of skin samples in addition to cloacal swabs could be valuable, as some bacteria species are more abundant in that anatomical part. 

The results of this study highlight the risk that aquatic turtle populations in the Canary Islands pose to human health, especially to animal handlers in close contact with these animals and their environment, as well as to immunocompromised individuals. Therefore, the use of protective equipment such as gloves and face masks when handling these reptiles is recommended, and direct contact with the animals or their surroundings with bare hands should be avoided.

## 5. Conclusions

Exotic freshwater turtle populations from the islands of Tenerife and Gran Canaria carry diverse bacteria relevant to human and veterinary health. Among the pathogens detected, Shiga-like toxin producer *E. coli*, non-tuberculous mycobacteria, and *S. aureus* pose the greatest threat to people, especially animal handlers, children, and the elderly; additionally, other animals can also become infected. Considering the preliminary results obtained in the study, further research is required to analyze additional bacterial species and serotypes in larger sample sizes in order to gain a better understanding of the health risks posed by exotic turtles in the Canary Islands.

## Figures and Tables

**Table 1:** Number of turtles studied by species and capture location.

	Location	Gran Canaria	Tenerife	Total
Species				
*Graptemys pseudogeographica*		2	0	2
*Mauremys* spp.		4	3	7
*Pseudemys peninsularis*		3	0	3
*Trachemys scripta*		17	13	30
Total		22	20	42

**Table 2:** Pathogenic bacteria isolated from freshwater turtles from Tenerife and Gran Canaria (Canary Islands, Spain).

Bacteria	Turtle Species	+/n (Prevalence; 95% CI)
*Campylobacter* spp.; n = 42	*Graptemys pseudogeographica*	0/2
	*Mauremys* spp.	0/7
	*Pseudemys peninsularis*	1/3 (33.3%; 0.8–90.6)
	*Trachemys scripta*	3/30 (10.0%; 2.1–26.5)
	Total	4/42 (9.5%; 2.7–22.6)
*Escherichia coli* (*stx*_1_, *stx*_2_ and *eae* genes); n = 42	*Graptemys pseudogeographica*	1/2 (50.0%; 1.26–98.7)
	*Mauremys* spp.	3/7 (42.9%; 9.9–81.6)
	*Pseudemys peninsularis*	0/3
	*Trachemys scripta*	10/30 (33.3%; 17.3–52.9)
	Total	14/42 (33.3%; 19.6–49.5)
*Listeria monocytogenes*; n = 36	*Graptemys pseudogeographica*	0/2
	*Mauremys* spp.	0/6
	*Pseudemys peninsularis*	0/3
	*Trachemys scripta*	0/25
	Total	0/36
*Mycobacterium* spp.; n = 19	*Graptemys pseudogeographica*	–
	*Mauremys* spp.	1/2 (50.0%; 1.26–98.7)
	*Pseudemys peninsularis*	1/1 (100%; 2.5–100)
	*Trachemys scripta*	9/16 (56.3%; 29.9–80.2)
	Total	11/19 (57.9%; 33.5–79.7)
*Pseudomonas* spp.; n = 19	*Graptemys pseudogeographica*	–
	*Mauremys* spp.	0/2
	*Pseudemys peninsularis*	0/1
	*Trachemys scripta*	2/16 (12.5%; 1.6–38.3)
	Total	2/19 (10.5%; 1.3–33.1)
*Salmonella* spp.; n = 42	*Graptemys pseudogeographica*	0/2
	*Mauremys* spp.	1/7 (14.3%; 0.4–57.9)
	*Pseudemys peninsularis*	3/3 (100%; 29.2–100)
	*Trachemys scripta*	9/30 (30.0%; 14.7–49.4)
	Total	13/42 (31.0%; 17.6–47.1)
*Staphylococcus* spp.; n = 42	*Graptemys pseudogeographica*	1/2 (50.0%; 1.3–98.7)
	*Mauremys* spp.	1/7 (14.3%; 0.4–57.9)
	*Pseudemys peninsularis*	1/3 (33.3%; 0.8–90.6)
	*Trachemys scripta*	6/30 (20.0%; 7.7–38.6)
	Total	9/42 (21.4%; 10.3–36.8)
*Vibrio* spp.; n = 36	*Graptemys pseudogeographica*	0/2
	*Mauremys* spp.	0/7
	*Pseudemys peninsularis*	1/3 (33.3%; 0.8–90.6)
	*Trachemys scripta*	1/30 (3.3%; 0.1–17.2)
	Total	2/36 (5.6%; 0.7–18.7)
*Yersinia enterocolitica*; n = 19	*Graptemys pseudogeographica*	–
	*Mauremys* spp.	1/2 (50.0%; 1.3–98.7)
	*Pseudemys peninsularis*	1/1 (100%; 2.5–100)
	*T. scripta*	6/16 (40.0%; 16.3–67.7)
	Total	8/19 (37.5%; 15.2–64.6)

+: number of infected animals, n: total of animals, 95% CI: 95% Clopper–Pearson confidence interval, –: no specimen was tested.

**Table 3:** Number of *E. coli* carrying virulence genes *eae*, *stx_1_*, and *stx_2_* isolates from freshwater turtles from Tenerife and Gran Canaria (Canary Islands, Spain).

	Turtle Species	*Graptemys* *pseudogeographica* (n = 2)	*Mauremys* spp. (n = 7)	*Pseudemys peinsularis* (n = 3)	*Trachemys scripta* (n = 30)	Total (n = 42)
Virulence Genes						
*eae*		0	0	0	6 (20.0%; 7.7–38.6)	6 (14.3%; 5.4–28.5)
*stx*_1_		0	1 (14.3%; 0.4–57.9)	0	3 (12.0%; 2.5–31.2)	4 (9.5%; 2.7–22.6)
*stx*_2_		1 (50%; 1.3–98.7)	2 (28.6%; 3.7–71.0)	0	4 (16.0%; 4.5–36.1)	7 (16.7%; 7.0–31.4)
Total		1 (50%; 1.3–98.7)	3 (42.9%; 9.9–81.6)	0	10 (43.3%; 25.4–62.6)	17 (40.5%; 25.6–56.7)

(Prevalence; 95% CI).

**Table 4:** Number of *Mycobacterium* spp. isolates in freshwater turtles from Tenerife and Gran Canaria (Canary Islands, Spain).

	Island	Gran Canaria (n = 5)	Tenerife (n = 14)	Total (n = 19)
Turtle Species				
*Graptemys pseudogeographica*		–	–	–
*Mauremys* spp.		–	1/2 (50.0%; 1.3–98.7)	1/2 (50.0%; 1.3 -98.7)
*Pseudemys peninsularis*		1/1 (100%; 2.5–100)	–	1/1 (100%; 2.5–100)
*Trachemys scripta*		2/4 (50.0%; 6.8–93.2)	7/12 (58.3%; 27.7–84.8)	9/16 (56.3%; 29.9–80.2)
Total		3/5 (60.0%; 14.7–94.7)	8/14 (57.1%; 28.9–82.3)	11/19 (57.9%; 33.5–79.7)

(Prevalence; 95% CI), –: no specimen was tested.

**Table 5:** Number of *Salmonella* spp. isolates detected in freshwater turtles from Tenerife and Gran Canaria islands (Canary Islands, Spain).

	Island	Gran Canaria (n = 26)	Tenerife (n = 16)	Total (n = 42)
Turtle Species				
*Graptemys pseudogeographica*		0/2	0	0/2
*Mauremys* spp.		0/4	1/3 (33.3%; 0.08–90.6)	1/7 (14.3%; 0.4–57.9)
*Pseudemys peninsularis*		3/3 (100%; 29.2–100)	0	3/3 (100%; 29.2–100)
*Trachemys scripta*		7/17 (41.2%; 18.4–67.1)	2/13 (15.4%; 1.9–45.4)	9/30 (30.0%; 14.7–49.4)
Total		10/26 (38.5%; 20.2–59.4)	3/16 (18.8%; 4.0–45.6)	13/42 (31.0%; 17.6–47.1)

(Prevalence; 95% CI).

**Table 6:** Number of *Staphylococcus* spp. isolates detected in freshwater turtles from Tenerife and Gran Canaria (Canary Islands, Spain).

	Island	Gran Canaria (n = 26)	Tenerife (n = 16)	Total (n = 42)
Turtle Species				
*Graptemys pseudogeographica*		1/2 (50.0% 1.3–98.7)	0	1/2 (50.0% 1.3–98.7)
*Mauremys* spp.		1/4 (25.0%; 0.6–80.6)	0/3	1/7 (14.3%; 0.4–57.9)
*Pseudemys peninsularis*		1/3 (33.3%; 0.8–90.6)	0	1/3 (33.3%; 0.8–90.6)
*Trachemys scripta*		4/17 (23.5%; 6.8–49.9)	2/13 (15.4%; 1.9–45.4)	6/30 (20.0%; 7.7–38.6)
Total		7/26 (26.9%; 11.6–47.8)	2/16 (12.5%; 1.6–38.3)	9/42 (21.4%; 10.3–36.8)

(Prevalence; 95% CI).

**Table 7:** Number of *Y. enterocolitica* isolates detected in freshwater turtles from Tenerife and Gran Canaria (Canary Islands, Spain).

	Island	Gran Canaria (n = 5)	Tenerife (n = 14)	Total
Turtle Species				
*Graptemys pseudogeographica*		–	–	–
*Mauremys* spp.		–	1/2 (50.0%; 1.3–98.7)	1/2 (50.0%; 1.3–98.7)
*Pseudemys peninsularis*		1/1 (100%; 2.5–100)	–	1/1 (100%; 2.5–100)
*Trachemys scripta*		1/4 (25.0%; 0.6–80.6)	5/12 (41.7%; 15.2–72.3)	6/15 (40.0%; 16.3–67.7)
Total		2/5 (40.0%; 5.3–85.3)	6/14 (42.9%; 17.7–71.1)	8/19 (42.1%; 20.3–66.5)

(Prevalence; 95% CI), –: no specimen was tested.

## Data Availability

All data obtained are included within the article.
